# Clinical Insights Into Pediatric Solid Pseudopapillary Neoplasms of the Pancreas

**DOI:** 10.7759/cureus.70655

**Published:** 2024-10-01

**Authors:** Atsushi Harada, Masashi Kurobe, Kazuaki Miyaguni, Tetsuro Sugihara, Sayuri Kaji, Daisuke Kanamori, Goki Uchida, Yuji Baba, Tomomasa Hiramatsu, Shinsuke Ohashi

**Affiliations:** 1 Pediatric Surgery, Kawaguchi Municipal Medical Center, Kawaguchi, JPN; 2 Pediatric Surgery, The Jikei University School of Medicine, Tokyo, JPN

**Keywords:** laparoscopic resection, postoperative outcome, solid pseudopapillary tumor of the pancreas, surgical enucleation, traumatic injury

## Abstract

Background: Solid pseudopapillary neoplasms (SPNs) of the pancreas are rare tumors with low malignant potential that usually occur in young girls and women. This study investigated the treatment experiences and outcomes after surgery for pediatric SPNs in our institutions as pediatric case series of solid SPNs are few, and long-term follow-up is also limited.

Methods: We retrospectively reviewed the outcomes of nine patients diagnosed with SPNs who underwent surgery in our three hospitals (The Jikei University Hospital, The Jikei University Kashiwa Hospital, and Kawaguchi Municipal Medical Center) between 2001 and 2023.

Results: All nine patients who underwent surgery were girls. Their ages ranged from 8 to 15 years (median: 10 years). The location of the tumor was at the pancreatic head, body, and tail in five, one, and three patients, respectively. Enucleation, pancreaticoduodenectomy, and laparoscopic distal pancreatectomy (LDP) were performed in four, two, and three patients, respectively. Regarding postoperative complications, a pancreatic fistula was detected in six patients, with three and three patients having grades A and B fistulas, respectively; two patients required percutaneous drainage, and one patient required endoscopic ultrasonography (EUS)-guided transgastric drainage. The follow-up period ranged from six to 261 months (median: 97 months). At the final follow-up, all nine patients were alive without recurrence.

Conclusion: SPNs of the pancreas are incidentally diagnosed by diagnostic workups following trauma in children more frequently compared to adults. Additionally, the tumor resection by minimally invasive approaches, such as enucleation, or laparoscopic procedures results in a good prognosis in some cases.

## Introduction

Solid pseudopapillary neoplasms (SPNs) of the pancreas are rare tumors with low malignant potential that usually occur in young girls and women [1]. Although pediatric pancreatic tumors are rare, SPNs are one of the most frequent neoplasms of the pancreas in childhood [2,3]. Complete surgical resection of an SPN is necessary for a favorable prognosis. Law et al. reported that SPN resection resulted in a mortality rate of less than 2% and that more than 95% of patients did not have recurrence [4]. However, SPNs may recur and metastasize to the liver.

While pediatric case studies of SPNs are limited and long-term follow-up is often lacking, SPNs in children are sometimes discovered following traumatic incidents, leading to urgent medical attention. Therefore, we reviewed the surgical techniques for SPN resection, its outcomes and complications, and long-term follow-up results in patients with SPNs based on the experience of three pediatric surgery institutions. 

## Materials and methods

We retrospectively reviewed the records of pediatric patients with SPNs who underwent surgery in three hospitals (The Jikei University Hospital, The Jikei University Kashiwa Hospital, and Kawaguchi Municipal Medical Center), which are connected to our Department of Pediatric Surgery; nine patients with SPNs who underwent surgery between 2001 and 2023 were included in the case series. The patients’ demographic characteristics, SPN onset, size, location, operative method, complications, pathological findings, and clinical outcomes were assessed. Continuous variables are reported as medians and ranges (minimum-maximum), whereas categorical variables are reported as frequencies and percentages. The postoperative complications were judged from the clinical records, radiological findings, and drainage fluid biochemistry. A pancreatic fistula was defined according to the recommendations of the International Study Group in Pancreatic Fistula [5].

Patients were evaluated using ultrasonography, computed tomography (CT), and magnetic resonance imaging (MRI) preoperatively. As for postoperative follow-up, all patients continued to visit the hospital regularly every three months to one year and follow-up periods were defined as the duration from the date of surgery to the last outpatient visit. Follow-up data were obtained by out-patient medical records and radiological findings such as CT or MRI retrospectively. This study was approved by the respective institutional review boards (No.31-182).

## Results

All nine patients were girls, and their ages at surgery ranged from eight to 15 years (median: 10 years). The location of the tumor was at the pancreatic head, body, and tail in five, one, and three patients, respectively. The clinical symptoms at diagnosis were abdominal pain due to tumor compression in four patients, abdominal pain after trauma in three patients, and a palpable mass in one patient. There was an asymptomatic case, which was incidentally detected by ultrasonography during postoperative follow-up for a choledochal cyst. Weight loss and jaundice were absent in all cases. The maximum diameters of all resected SPNs ranged from 2 cm to 10 cm (median: 5 cm). None of the patients had any evidence of liver or lymph node metastases before or after surgery. Endoscopic ultrasonography (EUS) was performed for preoperative diagnosis in Case 5. 

Patients were evaluated using ultrasonography, CT, and MRI preoperatively. Only one case (Case 3) was distinguished with malignant tumors. The surgical procedures employed for SPN resection were as follows: 1) laparoscopic distal pancreatectomy (LDP) with or without splenectomy; 2) pylorus-preserving pancreaticoduodenectomy (PpPD); and 3) enucleation. Fundamentally, enucleation via laparotomy was selected whenever possible to preserve function; however, pancreatic resection such as PpPD or DP was performed if vascular invasion or contact with the pancreatic duct was suspected.

As for postoperative complications, postoperative pancreatic fistulas (POPF) were detected in six patients; three had grade A and three had grade B. Percutaneous drainage was performed in two cases (Cases 1 and 2) and EUS-guided transgastric tube drainage was performed four months after surgery in Case 7. One patient developed a surgical site infection, which was managed with irrigation, drainage, and antibiotics (Case 2). The lengths of postoperative hospital stays ranged from nine to 62 days (median: 22 days), and the follow-up period ranged from six to 261 months (median: 97 months). At the last follow-up, all patients were still alive without recurrence and metastasis. The patient characteristics are summarized in Tables 1, 2.

**Table 1:** Summary of characteristics and clinical outcomes in our experiences. POPF: postoperative pancreatic fistulas; LDP: laparoscopic distal pancreatectomy

Clinical characteristics of patients with SPNs	n=9
Age at surgery (year)	10 (8-15)
Tumor size (cm)	5 (2-10)
Clinical symptoms	
Abdominal pain after trauma history	3
Abdominal pain without trauma	4
Palpable mass	1
Incidental detection	1
Tumor localization	
Head	5
Body	1
Tail	3
Operative method	
Enucleation	4
Pancreaticoduodenectomy	2
LDP (splenectomy)	1
LDP (spleen preserved)	2
Operative time (min)	369 (210-575)
Complications	
POPF grade A	3
Grade B	3
Surgical site infection	1
Hospital stay postoperation (days)	22 (9-62)
Recurrence or metastasis	0
Follow-up periods (months)	97 (6-261)

**Table 2:** Patients' characteristic details in our case series of SPNs. NK: not known; PD: pancreaticoduodenectomy; LDP: laparoscopic distal pancreatectomy; EN: enucleation; lap-SPDP: laparoscopic spleen preserved distal pancreatectomy; POPF: postoperative pancreatic fistulas

Case	Symptom	Age	Location	Preoperative diagnosis	Surgical procedure	Operation time (min)	Tumor size (cm)	Hospital stay (day)	POPF	Blood loss (cc)	Follow-up (months)
1	Abdominal pain	8	Tail	SPN	EN	210	8	61	Grade B	272	261
2	Abdominal pain after trauma	12	Head	SPN	EN	NK	7	20	Grade B	NK	260
3	Palpable mass	15	Head	Pancreatoblastoma	PD	540	5	61	Grade A	1733	236
4	Abdominal pain	13	Head	SPN	PD	575	10	33	Grade A	513	169
5	Abdominal pain	10	Head	SPN	EN	229	2	28	Grade A	1	97
6	Abdominal pain after trauma	12	Tail	SPN	LDP	294	3.6	11	-	40	90
7	Abdominal pain after trauma	10	Body	SPN	Lap-SPDP	563	6	11	Grade B	2	23
8	Abdominal pain	8	Head	SPN	EN	431	4	22	-	320	14
9	None	8	Tail	SPN	Lap-SPDP	306	3	9	-	450	6

**Figure 1:**
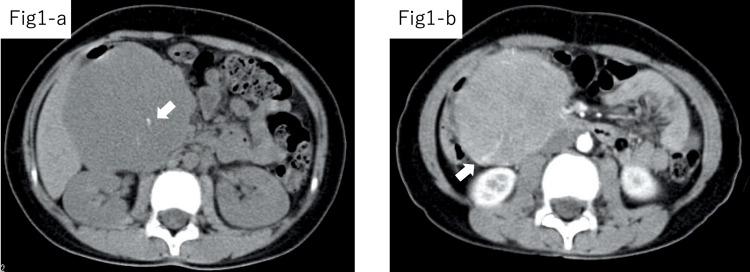
Preoperative CT findings in Case 3. CT revealed microcalcifications in the tumor (1-A, white arrow) and contrast enhancement in the arterial phase (1-B, white arrow) in Case 3. CT: computed tomography

In Case 3, the preoperative diagnosis was pancreatoblastoma. The CT revealed microcalcifications in the tumor with contrast enhancement in the arterial phase (Figure 1).

**Figure 2:**
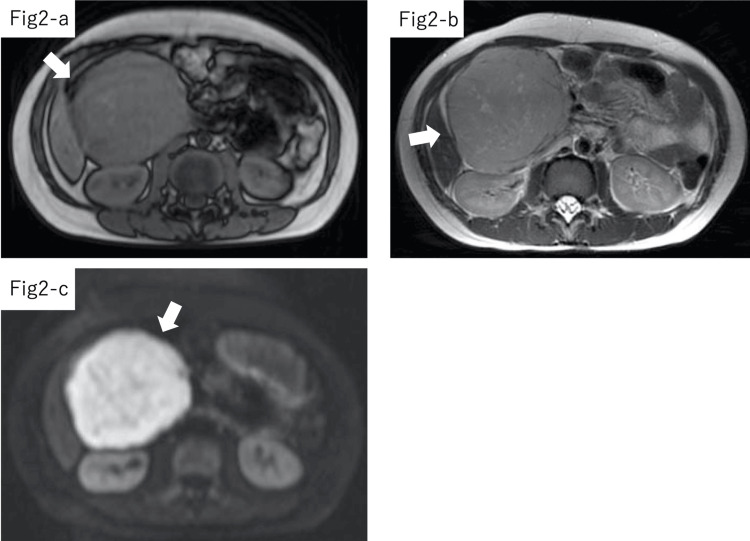
Preoperative MRI findings in Case 3. MRI confirmed the presence of a well-demarcated mass with a solid component of the same intensity as muscle on T1-weighted images (2-A, white arrow), slightly high intensity on T2-weighted images (2-B, white arrow), and extremely high intensity with homogenous enhancement on diffusion-weighted images (2-C, white arrow) in Case 3. MRI: magnetic resonance image

MRI confirmed the presence of a well-demarcated mass with a solid component of similar intensity as muscle on T1-weighted images, slightly high intensity on T2-weighted images, and extremely high intensity with homogenous enhancement on diffusion-weighted images (Figure 2), which is not contradictory to the findings of a pancreatoblastoma or malignancies such as a neuroendocrine tumor or germ cell tumor.

Thus, it was difficult to diagnose the tumor preoperatively until complete resection was performed so the patient was treated with three cycles of chemotherapy consisting of cisplatin and doxorubicin before complete tumor resection by PpPD. Chemotherapy was almost ineffective for the tumor volume; however, ultrasonography showed that intra-tumoral blood flow was decreased. The histopathological examination of the resected specimen confirmed SPN.

In Case 5, the preoperative placement of an endoscopic naso-pancreatic drainage stent (NPDS) was performed to avoid surgical injury of the pancreatic duct by enucleation reported by Tanaka et al. [6].

As for pathological and immunohistochemical characteristics, all resected specimens stained with hematoxylin and eosin showed small uniform cells with ovoid nuclei and eosinophilic granules arranged in sheets with pseudopapillary growth and hemorrhagic pseudo-cystic structures in various proportions, which are typical findings in SPNs.

Tumor capsule formation was noted in all patients except Case 3. All patients had pathologically negative resection margins. Meanwhile, the immunohistochemical staining results varied: beta-catenin was positive in five patients (100%; 5/5), vimentin was positive in seven patients (100%; 7/7), CD10 and CD56 were positive in six patients (100%; 6/6), alpha-antitrypsin was positive in six patients (100%; 6/6), synaptophysin was positive in three patients (60%; 3/5), and chromogranin A was positive in two patients (33%; 2/6). 

## Discussion

An SPN was first described by Frantz in 1959 as a “papillary tumor, benign and malignant” [7]. Since then, it has been called “Frantz’s tumor,” “solid and cystic tumor of the pancreas,” “adenocarcinoma of the pancreas of childhood,” “papillary-cystic tumor,” and “solid and papillary epithelial neoplasm.” In 1996, the World Health Organization (WHO) classification of pancreatic tumors termed the condition as “solid pseudopapillary tumors” of the pancreas [8]. In 2010, the WHO updated the disease as "solid pseudopapillary neoplasm of the pancreas" abbreviated as SPN.

SPNs usually affect young women in the second or third decades of life; however, about 20-25% of cases are seen in pediatric age groups [1]. The largest retrospective case series about SPNs in all age groups from a single institution was from China (n=243) in 2019, while multicenter cohort studies have been performed in the United States (n=721) in 2006 and in Japan (n=288) [1,9,10]. On the other hand, limited literature is available on the pediatric population. 

Then we investigated and reviewed previous case series involving four or more patients regarding pediatric SPNs written in English; a literature search was performed using PubMed. The largest pediatric retrospective review of 66 patients from Korea was reported in 2019, and they evaluated the indications for enucleation of SPNs [11]. Additionally, a retrospective review of 43 patients data from the Italian Pediatric Rare Tumor Registry in 2022, a retrospective analysis of 38 patients from the German Registry for Rare Pediatric Tumors in 2023, a multicenter retrospective study of 23 patients from 5 tertiary medical centers from Korea in 2006, a single-center experience of 18 patients from China in 2022, a review of 11 patients from Italy in 2011, a study involving 13 cases from the Czech Republic in 2012, a study that compared 15 pediatric SPN patients with adult counterparts from Hong Kong in 2008, and a number of case series regarding pediatric SPNs have also been published [12-25]. Hence, we summarized the issues involving four or more patients in Table 3. 

**Table 3:** Summary of previous pediatric SPN case series. NK: not known; R: recurrence; M: metastasis; D: death; PD: pancreaticoduodenectomy; DP: distal pancreatectomy; EN: enucleation

Author		Year	n	Female	Age (year)	Symptom			Tumor size (cm)	Prognosis	Follow-up period (months)	Location		Surgery		
						Abdominal pain	Palpable mass	Trauma related				Head	Body/Tail	PD	DP	EN
																	Yang [19]		1996	4	4	12.8 (12-14)	1	1	2	9 (6-10)	0R 0M 0D	28 (1-38)	1	3	1	2	1
Todani [20]		1998	4	4	13 (12-16)	2	2	0	6.6 (5-9)	1R 0M 0D	91 (17-148)	2	2	0	1	3
Jung [21]		1999	6	4	11 (8-13)	1	2	3	8.3 (6.5-10.5)	0R 0M 0D	70 (18-162)	5	1	5	1	0
Rebhand [22]		2001	4	4	15 (12-16)	3	1	1	10 (7-15)	1R 0M 0D	53 (6-144)	1	3	1	3	0
Choi [14]		2006	23	18	13 (10-15)	20	8	4	13 (10-15)	0R 1M 0D	62 (6-175)	7	16	6	15	2
Lee [18]		2008	15	13	12 (8-13)	5	9	NK	8 (3-14)	1R 0M 0D	67 (11-170)	10	5	10	4	0
Snajdauf [17]		2009	13	12	14 (9-17.5)	9	2	2	7 (5-15)	0R 0M 0D	103 (6-232)	7	6	1	6	0
Zampieri [16]		2011	11	9	NK (5-14)	6	2	3	NK (5-14)	0R 0M 0D	NK (NK-NK)	5	6	NK	NK	NK
Speer [23]		2012	11	11	14 (9-17)	8	1	2	5 (4-12)	1R 0M 0D	16 (6-71)	5	6	4	5	1
Laje [24]		2013	6	5	15 (11-18)	5	1	NK	6.8 (3-15)	0R 0M 0D	78 (6-180)	2	4	2	4	0
Ozcan [25]		2018	6	4	14 (13-16)	5	0	0	7.5 (5-10)	0R 0M 0D	71.5 (30-96)	3	3	3	2	0
Crocoli [12]		2018	43	35	13.2 (7-18)	26	4	NK	NK	1R 2M 0D	101 (0-204)	14	29	10	21	3
Cho [11]		2019	66	56	14.5 (NK-NK)	NK	NK	NK	6.1 (NK-NK)	3R 0M 0D	511.2 (NK-NK)	26	40	NK	NK	15
Maimaijiang [15]		2022	18	15	11.7 (8-11)	7	4	2	6.7 (3.2-11)	0R 0M 0D	NK (NK-NK)	7	11	4	6	3
Jentzsch [13]		2023	38	31	14.5 (13-15.3)	20	7	5	8 (5.3-9.6)	0R 0M 0D	23 (11-39)	14	22	10	21	1
Present case		2024	9	9	10 (8-15)	7	1	3	5 (2-10)	0R 0M 0D	97 (6-261)	5	4	2	3	4

The clinical symptoms of SPNs are related to the location and size of the mass, which compresses the adjacent organs. Papavramidis et al. reported that approximately 15.5% of the 718 patients with SPNs in their study were asymptomatic at presentation, whereas others presented with abdominal pain (45.6%) or a palpable mass (34.8%) [1]. Our case series review revealed that abdominal pain (57.9%; 117/202) and a palpable mass (21.8%; 44/202) were usually present at diagnosis, which are similar to those of previous studies of SPNs in all age groups. Our case series revealed abdominal pain (77.8%; 7/9) and a palpable mass (11.1%; 1/9), and abdominal pain is the largest symptom. The reason why the palpable mass was less than in other series was that the tumor size was relatively smaller than in other case series reviews.

Interestingly, a diagnosis of SPN was made after a traumatic episode in 17.4% (24/138) of patients in our pediatric summary, and our case series also revealed 33.3% (3/9). In contrast, Papavramidis et al. reported that 3.1% (20/643) of SPN patients were diagnosed following trauma in all age groups. Incidental detection after abdominal trauma may be a specific primary onset for pediatric patients with SPNs. 

Making a preoperative diagnosis of SPN is challenging as differentiating it from a pancreatic pseudocyst or other pancreatic malignancies in children is difficult due to their overlapping radiological features, and we also had difficulty in making a preoperative diagnosis in Case 3 [26]. Recently, endoscopic ultrasound-guided fine needle aspiration (EUS-FNA) is sometimes performed preoperatively, with Hanada et al. reporting an accuracy of 89% for diagnosing SPNs [10]. Additionally, immunohistochemical staining is also useful to diagnose and differentiate SPNs from other tumors such as pancreatic neuroendocrine tumors, pancreatic acinar cell carcinoma, and pancreatoblastoma. Lin et al. showed that beta-catenin, E-cadherin, CD10, vimentin, and chromogranin were the most effective stains to confirm the presence of SPNs [27]. In our case series, all cases stained with beta-catenin, vimentin, and CD10 were positive, which was generally consistent with previous reports [24,25]. 

Surgical resection is the primary treatment for SPNs. Complete resection should be performed as incomplete resections are frequently associated with local recurrence and poor prognosis. Minimally invasive surgery, such as enucleation or local resection, can be performed for selected cases as lymph node metastasis or invasion occurs in only 0-0.4% of cases [1,8]. Cho et al. reported that enucleation is a safe and effective surgical procedure that can preserve pancreatic function in children; however, it requires longer fasting times and hospital stays for recovery when compared with pancreatectomy [11]. Preoperative NPDS placement may ensure safe enucleation, as shown in Case 5. For the past 10 years, lap distal pancreatomy (LDP), which is minimally invasive, has also been performed for SPNs. Nanoong et al. reported that LDP results in a shorter duration of hospital stay than the conventional open method in children [28]. In our study, LDP was performed in three patients with SPNs of the pancreatic body and tail, both of whom were discharged early.

SPNs have low malignant potential and grow slowly. The prognosis of patients with SPNs has been reported by several studies. Yepuri et al. revealed that the recurrence rate of SPNs after surgical resection is approximately 2%; however, the five- and 10-year recurrence rates may be up to 71% and 4%, respectively [29]. In our summary of pediatric cases, 3.0% (8/268) of the patients had local recurrence, while 1.1% (3/268) had liver metastasis, with both values comparable to those from other studies. As it remains unclear which among the prognostic factors, including sex, tumor size, the solid component of the tumor, perineural invasion, infiltration into the adjacent tissue, and vascular invasion, are related to malignancy, patients should undergo regular annual clinical examinations and follow-ups for more than 10 years [30]. There were no recurrence and metastasis cases in our case series, and we believe that complete resection with clear margins, along with a regular postoperative follow-up, may have contributed to achieving these favorable outcomes.

There were several limitations in our study, which included the retrospective design and small sample size. Accumulating additional data and undertaking future studies with a larger population and a higher level of evidence involving multiple facilities are required.

## Conclusions

We summarized nine cases of SPNs who underwent surgical treatment in three institutions that resulted in favorable prognoses. We consider it important to select the appropriate surgical procedure such as DP, pancreaticoduodenectomy, or enucleation for each case in order to perform complete resection and prevent local recurrence. Careful long-term follow-ups are also required for pediatric patients, and the SPN resection results in a good prognosis in some cases. Additionally, we report SPNs are incidentally diagnosed by diagnostic workups following trauma in children more frequently than adults from previous case series reviews.
